# Kombucha as a Potential Active Ingredient in Cosmetics—An Ex Vivo Skin Permeation Study

**DOI:** 10.3390/molecules29051018

**Published:** 2024-02-26

**Authors:** Karolina Jakubczyk, Anna Nowak, Anna Muzykiewicz-Szymańska, Łukasz Kucharski, Kinga Szymczykowska, Katarzyna Janda-Milczarek

**Affiliations:** 1 Department of Human Nutrition and Metabolomics, Pomeranian Medical University in Szczecin, 24 Broniewskiego Street, 71-460 Szczecin, Poland; karolina.jakubczyk@pum.edu.pl (K.J.); kinga.szymczykowska@pum.edu.pl (K.S.); katarzyna.janda.milczarek@pum.edu.pl (K.J.-M.); 2 Chair and Department of Cosmetic and Pharmaceutical Chemistry, Pomeranian Medical University in Szczecin, Powstańców Wielkopolskich Ave. 72, 70-111 Szczecin, Poland; anna.nowak@pum.edu.pl (A.N.); lukasz.kucharski@pum.edu.pl (Ł.K.)

**Keywords:** green tea kombucha, black tea kombucha, ex vivo skin permeation, antioxidant activity, phenolic acid, caffeine

## Abstract

Kombucha is a non-alcoholic beverage, that is increasingly used in the cosmetic industry. The available literature reports the positive effects of kombucha on the skin, in particular its antioxidant action. However, there is a lack of information on skin permeation and the accumulation of active ingredients showing such effects. Skin aging is largely dependent on oxidative stress, therefore in our study we assessed the ex vivo permeation of two types of kombucha (green and black tea) through porcine skin. The antioxidant activity (DPPH, ABTS, FRAP methods) and total polyphenol content of these extracts were determined before and after permeation testing. Moreover, the content of selected phenolic acids as well as caffeine was assessed. Skin permeation was determined using a Franz diffusion cell. The antioxidant activity of both Kombuchas was found to be high. In addition, gallic acid, chlorogenic acid, protocatechuic acid, coumaric acid, m-hydroxybenzoic acid, and caffeine were identified. A 24-h ex vivo study showed the permeation of some phenolic acids and caffeine and their accumulation in the skin. Our results confirm the importance of studying the skin permeation of what are still little known ingredients in cosmetic preparations. Evaluation of the accumulation of these ingredients can guarantee the efficacy of such preparations.

## 1. Introduction

In recent years, there has been growing interest in using as many raw materials of natural origin as possible in cosmetic products. New technologies for the use of plant raw materials are increasingly being sought, so that they are characterized by the greatest possible amount of biologically active compounds [1]. There is also growing interest in the use of plant ferments in cosmetic preparations. The microbial strains used in the fermentation process influence the production of additional beneficial compounds, such as amino acids, proteins, and ceramides [2]. In addition, due to their high content of antioxidant substances, they have a high antioxidant activity, which is very beneficial in the context of skin application [3]. One ingredient that is becoming increasingly popular in cosmetic preparations is kombucha drink. Kombucha is a non-alcoholic fermented tea-based beverage that has been known for thousands of years and originated in East Asia. Around 414 AD, Dr Kombu brought it from Korea to Japan, where it was appreciated for its properties in relieving digestive ailments. In the 20th century, it was brought to Eastern Europe from Russia, where it was known as “tea acid” [4,5]. The name kombucha is derived from Dr Kombu, and the cha part refers to tea in Japanese. It is also known as Chinese mushrooms or Japanese mushrooms [6].

The preparation of kombucha involves the addition of 6–8 g of tea leaves and 60–100 g of sugar to a liter of water at a temperature of 70–95 °C. For the production of kombucha, sucrose is usually used, which is the energy source needed for the fermentation processes. After 10–15 min, the brew is strained and cooled, and then about 100 mL of the sourdough starter from the previous fermentation is added, as well as the SCOBY, a symbiotic culture of bacteria and yeast, which should make up about 10% of the total volume of the drink [7]. For 7–14 days, the drink should be left at room temperature without access to light. The solution is then separated from the newly formed tea fungus and filtered through cloth or gauze, then stored under refrigeration [4]. Three types of fermentation take place during kombucha production: lactic, alcoholic, and acetic. The SCOBY micro-organisms, consisting of acetic acid bacteria (AAB), lactic acid bacteria (LAB), and yeast, are responsible for their initiation [6,8].

The bacteria *Acetobacter xylinum* and the yeast, a representative of the osmophilic group, *Schizosaccharomyce spombe* are the main components of SCOBY. These bacteria are responsible for the production of acetic acid as a result of acetic fermentation, where the substrate is ethyl alcohol. Yeast, on the other hand, is responsible for the induction of sucrose breakdown, in which glucose is formed. This glucose is then the substrate for lactic and alcoholic fermentation [9]. Glucose is also oxidized to glucuronic acid and gluconic acid by *Acetobacter xylinum*, and during this process cellulose is synthesized, forming a dense biofilm that is the main component of the tea fungus [10]. The ongoing fermentation processes are influenced by many factors, including temperature, pH, oxygen content, and dissolved CO_2_. Changes in these parameters may affect the fermentation rate, yield, organoleptic properties, nutritional quality, and physico-chemical properties [11].

The drink’s sour taste is determined by its organic acid content (gluconic, tartaric, malic, citric), particularly acetic acid [12]. Kombucha is also a source of vitamins, e.g., E, K, and B, and minerals such as potassium, manganese, and fluoride. In addition, the drink is rich in amino acids, particularly theanine, and polyphenolic compounds, among other flavonoids [7]. Due to the high content of these ingredients, kombucha has proven antioxidant, antibacterial, antidiabetic, immune-stimulating, and cholesterol-reducing effects and stimulates liver detoxification [13].

Due to its health-promoting properties, kombucha is attracting increasing interest, especially in the cosmetic industry. It is increasingly being proposed as an interesting ingredient in cosmetic preparations, with moisturizing, anti-aging, or anti-inflammatory effects [14,15,16,17]. Previous reports have indicated that topically applied kombucha has beneficial effects on the skin. For example, kombucha prepared from the leaves of *Rubus fruticosus*, *Vaccinum myrtillus*, *Ribes nigrum*, or *Fragaria vesca* showed strong anti-aging properties through its ability to inhibit the activity of metalloproteinases such as collagenase and elastase. In addition, the authors observed a positive effect of all the ferments analyzed on skin hydration and pH [15]. Some compounds, including polyphenols, proteins, and carbohydrates, which are also present in kombucha, are responsible for the moisturizing effect of plant extracts. Moisturizing properties are demonstrated by both low and high molecular weight compounds. Low-molecular-weight compounds penetrate the deeper layers of the skin and keep water. High-molecular-weight compounds have a much weaker penetration ability, but thanks to their occlusive properties, they limit trans-epidermal water loss (TEWL) [14]. Numerous studies have also demonstrated the low toxicity of kombucha to fibroblasts and keratinocytes [15,17], as well as the absence of irritation when applied to the skin [16]. According to some authors, cosmetic preparations containing kombucha were characterized by a higher bio-availability of valuable secondary metabolites. In addition, cosmetic preparations containing bio-ferments in their composition are increasingly classified as natural and environmentally friendly cosmetics [2]. Kombucha owes its pharmacological effects, among other factors, to bioactive compounds such as phenolic acids or caffeine [18]. These ingredients mainly exhibit antioxidant effects in preparations applied to the skin. However, there is a lack of information on their permeation through the skin, as well as their accumulation in the skin. Therefore, our study aimed to evaluate the chemical composition, the antioxidant activity of kombucha, and skin permeation, as well as accumulation in the skin of selected secondary metabolites showing antioxidant activity. Such a study helps to assess to what extent the active substances contained in kombucha may be useful, not only to protect the upper layers of the skin, but also its deeper layers, against oxidative stress.

## 2. Results

### 2.1. Antioxidant Activity and pH

Kombucha made from both green and black tea has a high antioxidant potential (Table 1). The beverage prepared from green tea (GTK) had a higher antioxidant potential when evaluated using the ABTS method than the black tea kombucha (BTK). The antioxidant activity of GTK was 93.88% RSA and 3.43 mmol trolox·dm^−3^, while that of BTK displayed 71.01% RSA and 2.59 mmol trolox·dm^−3^. The antioxidant activity evaluated by the DPPH of both kombuchas was at a similar level of 87%. The antioxidant potential measured by the FRAP method was 8.87 mmol FeSO_4_·dm^−3^ for GTK and 3.36 mmol FeSO_4_·dm^−3^ for BTK. The Folin–Ciocalteu method showed a similar polyphenol content of 195.84 mg GA·dm^−3^ for GTK and 188.13 mg GA·dm^−3^ for BTK. Statistically significant differences between the kombuchas were recorded for the ABTS and FRAP measurements (Table 1). The tested kombuchas were characterized by an acidic pH. GTK had a higher pH of 3.0, while BTK had a lower pH of 2.7.

### 2.2. HPLC Analysis

**Figure 1:**
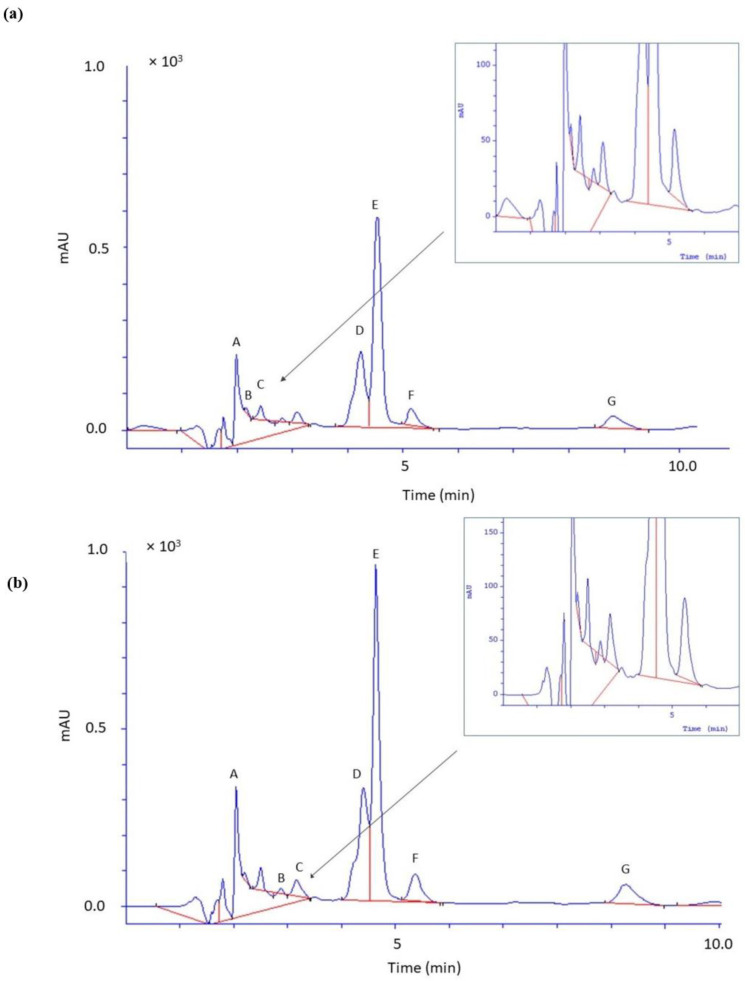
Chromatogram of phenolic acids and caffeine identified in green (**a**) and black (**b**) tea kombucha. A—gallic acid; B—protocatechuic acid; C—chlorogenic acid; D—caffeic acid; E—caffeine, F—m-hydroxybenzoic and G—coumaric acid. Kombucha samples were diluted twice before HPLC analysis.

Six phenolic acids and caffeine were identified in the kombucha. The contents of the individual acids were as follows for GTK: gallic acid (32.84 mg·dm^−3^), caffeic acid (20.12 mg·dm^−3^), coumaric acid (12.31 mg·dm^−3^), m-hydroxybenzoic acid (10.96 mg·dm^−3^), chlorogenic acid (5.13 mg·dm^−3^), protocatechuic acid (3.43 mg·dm^−3^); while in BTK: gallic acid (49.22 mg·dm^−3^), caffeic acid (30.50 mg·dm^−3^), coumaric acid (19.29 mg·dm^−3^), m-hydroxybenzoic acid (19.15 mg·dm^−3^), chlorogenic acid (7.60 mg·dm^−3^), protocatechuic acid (5.01 mg·dm^−3^). Among the identified phenolic acids, the lowest content was found for protocatechuic acid, and the highest for gallic acid. Additionally, BTK was characterized by a higher content of all identified compounds, and these differences were statistically significant. Both samples had high amounts of caffeine, with the content being higher in BTK (165.49 mg·dm^−3^) than in GTK (102.87 mg·dm^−3^) (Figure 1 and Table 2).

### 2.3. Ex Vivo Skin Permeation 

#### 2.3.1. Antioxidant Activity and Total Polyphenol Content

Table 3 shows the antioxidant activity and total polyphenol content in both types of kombucha applied to the skin, in the extract prepared from the skin used for permeation tests, and in the acceptor fluid after 24-h permeation. In general, all samples showed antioxidant activity. Samples obtained from the skin after 24-h permeation were characterized by significantly higher antioxidant activity and TPC in the case of the BTK compared to samples prepared from the skin after GTK permeation. For the BTK, this was 0.40 mmol trolox·dm^−3^ for the DPPH method and 104.02 mg GA·dm^−3^ for the TPC. The percentage of accumulation of compounds with antioxidant potential in the skin was as high as 48.19% for the DPPH method and 55.29% for the TPC. The values for GTK were 0.31 mmol trolox·dm^−3^ and 88.27 mg GA·dm^−3^, representing, respectively, 37.80% and 45.07% accumulation in the skin relative to the antioxidant potential of kombucha applied to the skin. The acceptor fluid taken after the permeation test showed higher antioxidant activity and total polyphenol content for BTK, 0.07 mmol trolox·dm^−3^ (DPPH method) and 28.28 mg GA·dm^−3^ (TPC). Slightly lower activity was exhibited by the acceptor fluid taken after GTK application, 0.05 mmol trolox·dm^−3^ and 24.60 mg GA·dm^−3^, respectively (Table 3).

#### 2.3.2. Phenolic Acid and Caffeine Content

**Figure 2:**
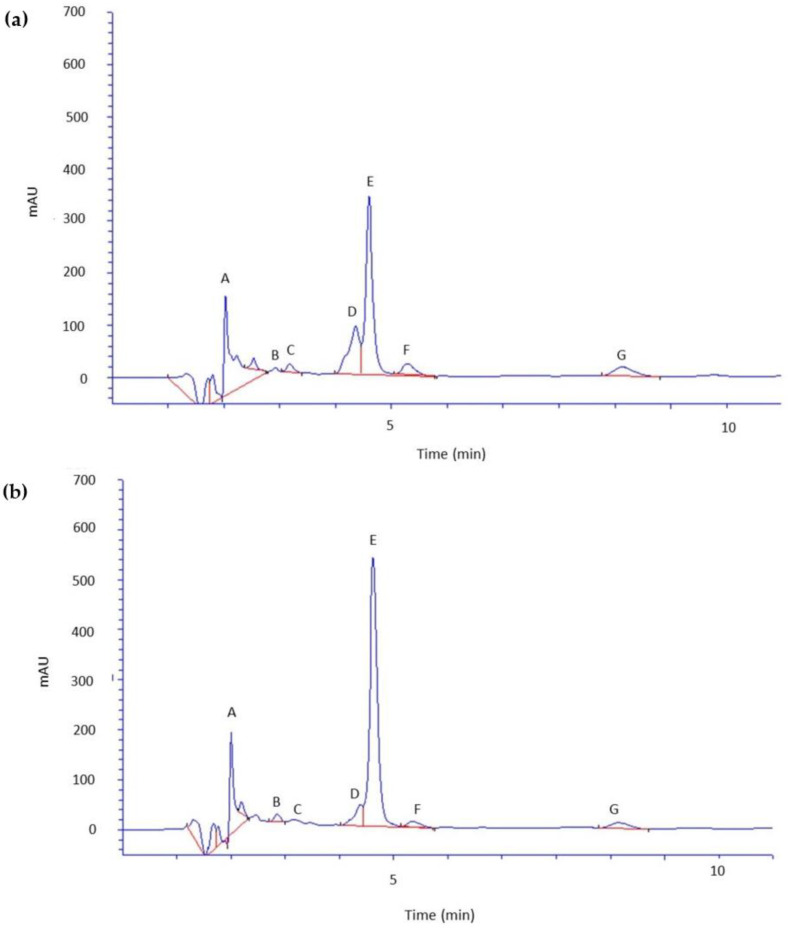
Chromatograms of compounds identified in skin extract after 24-h permeation: (**a**) skin extract after permeation of green tea kombucha; (**b**) skin extract after permeation of black tea kombucha; A—gallic acid; B—protocatechuic acid; C—chlorogenic acid; D—caffeic acid; E—caffeine, F—m-hydroxybenzoic acid, and G—coumaric acid. Kombucha samples were diluted twice before HPLC analysis.

**Figure 3:**
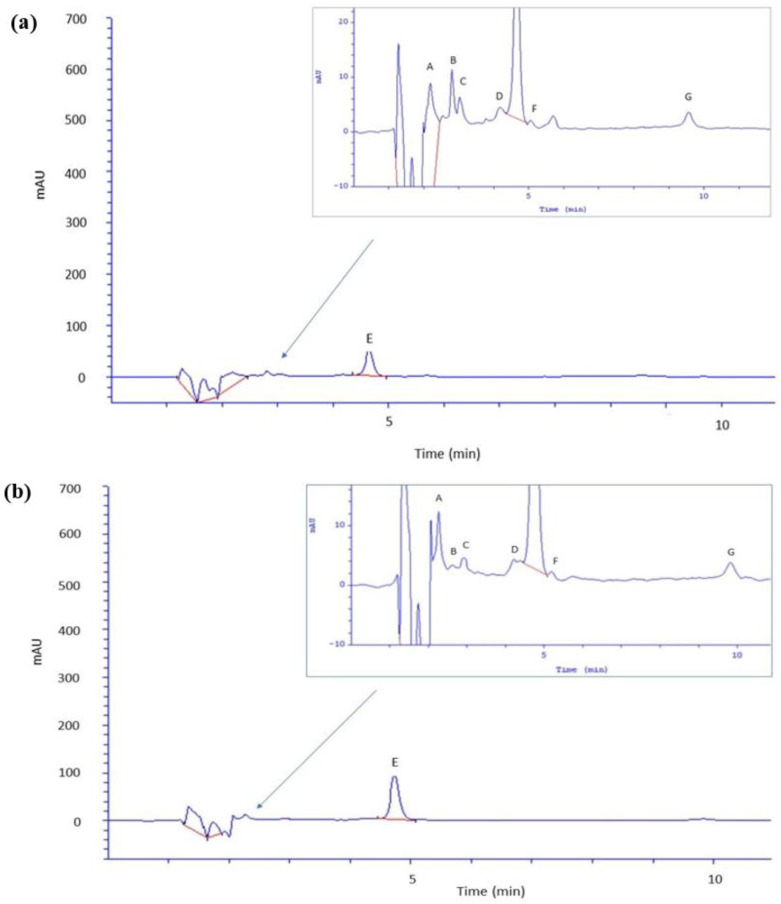
Chromatograms of compounds identified in acceptor fluid after 24-h permeation: (**a**) acceptor fluid after 24-h permeation of green tea kombucha (**b**) acceptor fluid after 24-h permeation of black tea kombucha; A—gallic acid; B—protocatechuic acid; C—chlorogenic acid; D—caffeic acid; E—caffeine, F—m-hydroxybenzoic acid, and G—coumaric acid. Kombucha samples were diluted twice before HPLC analysis.

Table 4 summarizes the content of selected phenolic acids and caffeine in the skin and the acceptor fluid collected after 24-h permeation of green and black tea kombucha, whereas Figure 2 shows the HPLC chromatogram of skin extract after permeation of GTK (a) and BTK (b) while Figure 3 shows acceptor fluid after 24-h permeation of GTK (a) and BTK (b).

By analyzing the extract obtained after skin extraction, it was found that all the analyzed compounds permeate the skin. Caffeine accumulates in the skin in the highest amounts. Its content was 304.84 µg·g^−1^ for GTK and 449.09 µg·g^−1^ for BTK. Of the tested phenolic acids, gallic acid was the compound most accumulated in the skin in the case of GTK–123.26 µg·g^−1^, while in the case of BTK, the highest concentration of the analyzed phenolic acids was found for m-hydroxybenzoic acid (143.59 µg·g^−1^). The accumulation of m-hydroxybenzoic acid in the skin in the case of GTK remained at a much lower level and amounted to 29.05 µg·g^−1^ (27.13%). Gallic acid from BTK also accumulated in large amounts, and its concentration was at a similar level to that in GTK–126.87 µg·g^1^ (26.44%).

When analyzing the percentage of accumulation of the tested phenolic acids in the skin, in the case of GTK the highest value was recorded for gallic acid (38.47%). In the case of BTK, the highest percentage of accumulation was found for m-hydroxybenzoic acid (76.95%). The accumulation in the skin of chlorogenic acid was 12.31 µg·g^−1^ (GTK) and 3.19 µg·g^−1^ (BTK) and this was the compound that accumulated to the least extent. The lowest percentage of accumulation in the skin was found for protocatechuic acid (17.09%) in the case of GTK, while in the case of BTK it was found for chlorogenic acid (4.49%). The accumulation of protocatechuic acid in the skin was at a similar level in the case of both kombuchas (28.31 µg·g^−1^ for GTK and 28.07 ± 1.21 µg·g^−1^ for BTK). Caffeic and coumaric acid accumulated in the skin in larger amounts after GTK application. Their content in the case of GTK was 42.60 µg·g^−1^ (21.75%) and 30.88 µg·g^−1^ (25.75%), respectively, and for BTK 19.23 µg·g^−1^ (6.73%) and 20.26 µg·g^−1^ (11.25%).

Among the tested compounds, the highest concentration in the acceptor fluid was found for caffeine. Its content was 36.01 µg (4.38%) for GTK and 62.68 µg (4.73%) for BTK. In the case of phenolic acids, for GTK, protocatechuic acid, m-hydroxybenzoic, and coumaric acid were present in the highest amount in the acceptor fluid (15.87 µg, 8.50 µg, and 5.93 µg, respectively). For BTK, the highest contents of acceptor were found for gallic acid (6.50 µg), protocatechuic acid (6.46 µg), and coumaric acid (5.74 µg). The compound whose content in the acceptor fluid was lowest was caffeic acid in the case of GTK (2.02 µg), while for BTK it was chlorogenic acid (2.19 µg). In the case of BTK, caffeic and m-hydroxybenzoic acid permeated the skin at similarly low levels (2.29 µg and 2.23 µg, respectively). When assessing the percentage of permeability, in the case of BTK the highest values were found for m-hydroxybenzoic acid and chlorogenic acid (9.69% and 9.48%, respectively). In the case of BTK, these were coumaric acid (3.71%) and chlorogenic acid (3.61%). The lowest permeation percentage in the case of GTK and BTK was observed for caffeic acid (1.00% and 0.75%, respectively).

**Figure 4:**
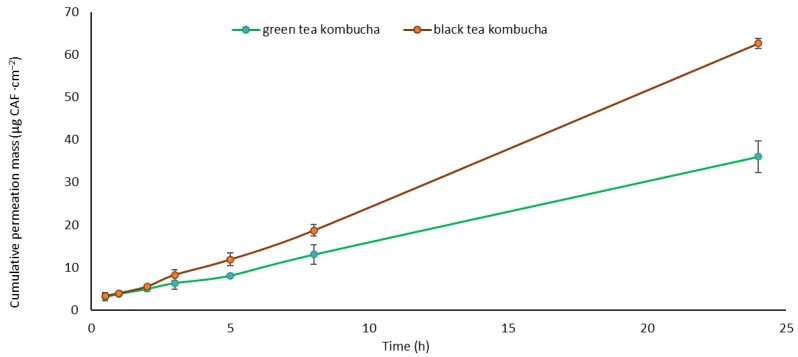
Time course of the cumulative mass of caffeine expressed in µg CAF·cm^−2^ during the 24-h permeation of kombuchas. CAF–caffeine, (n = 3).

Throughout the 24-h test, it was observed that caffeine was the only compound that permeated after the first hour of testing, while the other compounds were not detected in the acceptor fluid for the first eight hours, or their content was trace. Figure 4 shows the cumulative mass of caffeine contained in the acceptor fluid taken at specified intervals over the 24-h test. A significantly higher permeation of caffeine was observed at the fifth hour of the test for BTK compared to GTK (Figure 4). In the case of caffeine permeation during the 24-h study, cumulative mass of caffeine from GTK and BTK in acceptor fluid for the first two hours was similar. However, when analyzing the percentage of caffeine permeated through the skin in relation to the solution applied to the skin, the caffeine from GTK penetrated to a greater extent, 0.74% and 0.92% in the first and second hour, respectively. For comparison, these values for BTK were 0.25% and 0.30%, respectively.

Statistically significant differences in accumulation in the skin between the kombuchas were noted for chlorogenic acid, caffeic acid, caffeine, m-hydroxybenzoic acid, and coumaric acid. In contrast, statistically significant differences in compound content in acceptor fluid between the kombuchas were found for gallic acid, protocatechuic acid, chlorogenic acid, caffeine, and hydroxybenzoic acid.

## 3. Discussion

Kombucha is a fermented non-alcoholic tea-based beverage. It has many health-promoting properties, and therefore is used in cosmetic products as an aqueous phase in creams, tonics, or mists. In the present study, two types of kombucha were analyzed, BTK and GTK. The results show the high antioxidant potential, and a high content of phenolic acids and caffeine can permeate and accumulate in the skin.

The skin aging process is accelerated by oxidative stress, which occurs due to the overproduction of free radicals. These processes are induced by, for example, UV radiation or environmental pollutants. To minimize oxidative stress, it is important to provide antioxidants whose action, based on some mechanisms, leads to a reduction in oxidative stress and delays the skin aging process [19]. 

In our study, the analyzed kombuchas had a high antioxidant potential, which for both samples was about 87% RSA and 0.82–0.83 mmol trolox·dm^−3^ (DPPH method). For the ABTS method, the antioxidant activity was 71.01–93.88% ABTS radical inhibition and 2.59–3.43 mmol trolox·dm^−3^. For the FRAP method, the antioxidant potential ranged from 3.36 to 8.87 mmol FeSO_4_·dm^−3^, while the polyphenol content measured by the Folin–Ciocalteu method was 188.13–195.84 mg GA·dm^−3^. The high antioxidant potential of the tested teas is confirmed by other researchers [7,20,21,22]. Jakubczyk et al. [7] and Jayabalan et al. [23] obtained an equal relationship; GTK had a higher antioxidant potential than BTK. In the study by Jakubczyk et al., the antioxidant potential of GTK was 94.61% DPPH radical inhibition, while that of BTK was 78.62% [7]. However, a study by Cardoso et al. showed a different relationship, and a higher antioxidant potential was observed in BTK than in GTK [21]. Saimaiti et al., investigating the antioxidant potential of kombucha from vine tea and sweet tea, obtained slightly higher results than in the present study. However, the teas were characterized by lower gallic acid content [24]. Zhou et al. also subjected black and GTK to analysis and the results of antioxidant potential are similar to those obtained in this study, as are gallic acid and caffeine content [25]. Kombucha stands out for its high antioxidant potential compared to other teas, as well as other beverages. The antioxidant potential and phytochemical composition of kombucha are influenced by many parameters, one of which is the type of tea, but equally important is the quality or concentration of the tea. Furthermore, it has been shown that the antioxidant potential of kombucha can depend on the culture period and the origin of the starter culture. The antioxidant potential also varies with fermentation time [22]. In addition, the ability of teas to reduce free radical scavenging is conditioned by the time the leaves are harvested and how long they are stored after harvesting [26].

The examined content of active substances in natural preparations can help assess their potential dermatological activity. One of the group of compounds that play a crucial role in the effects of preparations applied to the skin is phenolic acids. The phenolic acids are valuable compounds due to their, among others, antioxidant and anti-inflammatory properties. Another very important compound that plays a key role in anti-aging preparations is caffeine, belonging to the alkaloids. In our study, selected phenolic acids and caffeine were identified in both kombuchas. The compounds in the studied kombucha were characterized by high levels of accumulation and partial permeation through the skin, as well as high antioxidant potential. The high accumulation of compounds with antioxidant properties influences their antioxidant potential and associated anti-aging effects. In the present study, protocatechuic acid, m-hydroxybenzoic acid, and gallic acid accumulated to the greatest extent in the skin. These acids have a high antioxidant potential, which is important in cosmetic products. Gallic acid has specific properties in retarding lipid peroxidation, which is a result of oxidative stress in the skin [27]. In addition, protocatechuic acid shows a potential stimulating effect on collagen synthesis and has shown the ability to inhibit the effects of UVA radiation on the skin, resulting in reduced wrinkles [28]. A similar effect is shown by m-hydroxybenzoic acid, which reduces the effects of oxidative stress in the skin and therefore delays the skin aging process [29]. Furthermore, in addition to phenolic acids, a compound that has largely accumulated in the skin is caffeine, which is commonly used in cosmetic products. It can reduce oxidative stress, causes a decrease in fatty acids in the cells, making it effective in the fight against cellulite, and increases the micro-circulation of blood in the skin [30].

In the study by Nowak et al. [31], the degree of skin permeation of extracts of fireweed was investigated. However, they had a three times higher antioxidant potential than kombucha. Despite this large difference, the results from the extract after skin extraction and acceptor fluid are very similar for the extract after skin extraction measured by the DPPH method. Here, extracts with a concentration of 5% (5 g fireweed per 100 mL ethanol) were used. Kombucha, on the other hand, has a concentration of about 0.08% (6–8 g of raw material per 1 L of water), which is more than 10 times less. The aforementioned study also analyzed the content of phenolic acids (chlorogenic acid, gallic acid, and caffeic acid) accumulating in the skin and the acceptor fluid. Their content was higher in both, compared to kombucha. The only difference was in the content of caffeic acid, whose content in the acceptor fluid was higher in the BTK than in the fireweed extracts [31]. However, according to Bertges et al., a lower accumulation in deeper skin layers is sometimes more effective in cosmetology [32]. In the study by Makuch et al. [33], the antioxidant potential and skin permeation ability of eugenol were studied. This is a compound mainly extracted from cloves. It has antioxidant properties and is often used in cosmetic products. The antioxidant potential of the solution applied to the skin was 1.23 mmol trolox/dm^3^ as measured by the DPPH method, which was higher than that of the kombucha tested in this study, but the antioxidant potential in the solution after skin extraction and acceptor fluid was very close to our results. The same relationship was shown in the same study in analyses carried out using the Folin–Ciocalteu method. This means that kombucha, despite its lower antioxidant potential, accumulates in the skin in a similar amount to that of the well-known and frequently used eugenol [33]. 

In our study, a small percentage of skin penetration for the analyzed compounds was observed. The penetration of active substances is influenced by many factors, among which the lipophilicity of the compound applied to the skin plays a very important role. Since the intercellular cement of the stratum corneum consists mainly of lipids, lipophilic substances penetrate the stratum corneum much more easily than hydrophilic substances. The measure of a compound’s lipophilicity and affinity for the lipid phase is the logarithm decimal of the partition coefficient of the active substance in the octanol mixture and water (log P) [34]. For example, in the case of caffeine, very high accumulation in the skin was observed, but very low penetration. The high hydrophilic properties of caffeine (log P = −0.07) [35] are probably the reason for the low penetration through the skin layer, which is lipophilic. A similar tendency was also observed in the case of other compounds characterized by high hydrophilicity, such as gallic acid (log P = −0.7) [36] or caffeic acid (log P = −0.66) [37].

The vehicle of the substance is very important during penetration into the skin [32,38]. Depending on the vehicle, the same active substances may accumulate in the skin or penetrate more deeply. Lower permeation of antioxidant ingredients through the skin increases the antioxidant capacity of the stratum corneum, while increased transdermal permeation of active substances is required if the compounds are contained in transdermal preparations [39]. When analyzing the ability to penetrate and permeate active substances from topical preparations, including cosmetics, one should remember their safety in the context of possible permeation into the systemic circulation. One of the factors influencing the release and penetration of active ingredients from topically applied preparations is the selection of the vehicle and excipients. Some substances act as permeation enhancers or retardants. Examples of permeation enhancers are terpenes contained in essential oils (e.g., menthol), which increase the percutaneous permeation parameters [40]. Moreover, to confirm the safety of a cosmetic preparation, tests to assess the penetration and permeation through animal or human skin are performed, which constitute an acceptable alternative to in vivo tests [41]. Another way to assess the safety of cosmetics and prevent systemic toxicity is to conduct toxicokinetic and toxicodynamic studies [42]. Therefore, to prevent penetration into systemic circulation and systemic toxicity, it seems crucial to select the appropriate excipients and vehicles in cosmetic preparations and to check the finished preparations for their safety.

The pH value of cosmetic products has an impact on the condition of the skin. The lower pH of cosmetics has been proven to reduce water loss through the skin, support the skin barrier, and reduce the peeling of the epidermis [43]. In addition, skin cleansing products containing predominantly anionic surfactants can cause increased dryness and irritation of the skin [44].

However, there are some limitations to this study. The basic composition of Kombucha presented in the Table 5 has not been experimentally confirmed and comes from the product label. The tolerance limit for nutrients listed on the label is commonly used, so this composition should be treated as an approximate composition.

## 4. Materials and Methods

### 4.1. Materials

The study material consisted of two kombucha teas, black (BTK) and green (GTK), purchased at a Polish food market (Delikatna.bio, Gdańsk, Poland) (Table 5). The kombucha bacterium component belongs to the strains of *Acetobacter*, along with the presence of yeasts *Saccharomyces cerevisiae*. Each sample came from 3 different bottles and was tested in triplicate.

### 4.2. Methods

#### 4.2.1. HPLC Analysis

The concentration of selected compounds was determined by high-performance liquid chromatography (HPLC–UV, Knauer, Germany) according to the modified method described by Nowak et al. [31,45]. The separation was performed on a C18 column (125 mm × 4 mm) containing Eurospher 100-5. Our previous studies have shown that the use of a C18 column allows adequate separation of the analyzed compounds. The mobile phase consisted of acetonitrile, 1% acetic acid, and MeOH (10:90 by vol.), the flow rate was 1 mL/min, and 20 µL of the tested sample was injected into the column. Individual peaks were identified based on reference substances. The chromatographic peaks of the tested compounds were confirmed by comparing their retention times with reference compounds. Solutions with the following concentrations were used to prepare standard curves: 0.01; 0.005; 0.0025; 0.00125; 0.000625, and 0.0003125%. The correlation coefficient of the calibration curve was 0.9999 for gallic acid (y = 157,710x − 4.1682, tR–2.035 min); 0.999 for protocatechuic acid (y = 54,833x + 27,283, tR–2.869 min); 0.9997 for chlorogenic acid (y = 1,125,566x + 4.2187, tR–3.169 min); 1.000 for caffeic acid (y = 205,227x + 0.8061, tR–4.403); 0.9996 for m-hydroxybenzoic acid (y = 75,786x + 0.3788, tR–5.354 min); 0.9998 for coumaric acid (y = 54,367x + 0.061, tR–9.290 min), and 0.9983 for caffeine (y = 118,553x − 22.691, tR–4.637 min). Each sample was analyzed in triplicate.

#### 4.2.2. Evaluation of the Antioxidant Activity and Total Polyphenol Content

The antioxidant activity of kombucha was evaluated using the DPPH, ABTS, and FRAP methods, and the total polyphenol content using the Folin–Ciocalteu technique. Measurements of each sample were performed in three independent replicates. DPPH stable free radical scavenging activity was measured according to the methodology described by Nowak et al. [46]. Briefly, 150 μL of the kombucha sample was mixed with 2850 μL of 0.3 mM DPPH radical solution prepared in 96% *v*/*v* ethanol. The DPPH working solution was diluted with concentrated ethanol to obtain an absorbance of 1.00 ± 0.02 at a wavelength of 517 nm. Measurement of absorbance (λ = 517 nm) using 96% (*v*/*v*) ethanol as blank zero was performed after 10 min of incubation in the dark at room temperature. As a reference, 6-hydroxy-2,5,7,8-tetramethylchroman-2-carboxylic acid (trolox) was applied. The results were expressed as trolox equivalents (TEAC) in mmol trolox·dm^−3^ and %RSA (radical scavenging activity). 

The methodology for assessing ABTS radical scavenging activity was described previously [46]. Shortly, a 7 mM solution of ABTS (2,2′-azino-bis(3-ethylbenzothiazoline-6-sulfonic acid)) in a 2.45 mM aqueous solution of K_2_S_2_O_8_ was used as a stock solution. The stock solution was incubated for 24 h in the dark at room temperature and then, to obtain a solution with absorbance 1.00 ± 0.02 at 734 nm, was diluted with 50% (*v*/*v*) methanol. The test sample was prepared by mixing 2500 μL of ABTS solution and 25 μL of the kombucha sample. The absorbance at 734 nm was measured after a 6-min incubation of the samples at room temperature. The results were expressed as trolox equivalents (TEAC) in mmol trolox·dm^−3^ and %RSA. 

The ferric ion-reducing potential was assessed using the FRAP method described previously [46]. The working solution was obtained after mixing 1 volume of 10 mM TPTZ (dissolved in 40 mM HCl), 1 volume of 20 mM FeCl_3_, and 10 volumes of acetate buffer (pH 3.6), and 2320 µL of this solution was mixed with 80 µL of the kombucha. The absorbance was measured at 593 nm after 15-min of incubation. The results were expressed as mmol FeSO_4_·dm^−3^ of kombucha.

Total polyphenol content was determined with the Folin–Ciocalteu method, as described previously [46]. Briefly, 150 μL of the kombucha, 150 μL of Folin–Ciocalteu reagent (tenfold diluted with water), 1350 μL of 0.01 M sodium carbonate solution and 1350 μL of water were mixed. The samples were incubated for 15 min at room temperature and then their absorbance was measured at 765 nm. Gallic acid (GA) was applied as a standard, and results were expressed as gallic acid equivalents (GAE) in mg GA·dm^−3^. 

#### 4.2.3. Ex Vivo Skin Permeation Studies

Ex vivo skin permeation studies were performed using Franz diffusion cells (Phoenix DB-6, ABL and E-JASCO, Wien, Austria). The acceptor chamber with a volume of 10 mL was filled with PBS solution (pH 7.4), while the kombucha samples (1 mL) were placed in a donor chamber. Each donor chamber was closed with plastic caps to prevent excessive evaporation of the test sample. The contents of all acceptor chambers were mixed with a magnetic mixer at a speed of 350 rpm. During the analysis, the temperature in the diffusion chambers was maintained at 37.0 ± 0.5 °C. Since pig skin is characterized by similar permeability to human skin [47,48], in this experiment abdominal porcine skin purchased in the local slaughterhouse (Agrofirma Witkowo, Szczecin, Poland) was used. The fresh porcine skin was washed several times in PBS buffer pH 7.4. Then, using a dermatome, skin samples with a thickness of 0.5 mm were cut and divided into pieces of 2 cm × 2 cm. The prepared samples, wrapped in aluminum foil, were stored at −20 °C until the experiment was carried out, for no longer than 3 months. These storage conditions and times are safe in maintaining the skin’s barrier properties [49]. Immediately before experimenting, the skin samples were slowly thawed at room temperature for 30 min and hydrated with PBS buffer at pH 7.4 [50,51,52]. Undamaged pieces of skin of equal thickness were used for the experiment. The integrity of the skin was tested by checking its impedance using an LCRmeter4080 m (Conradelectronic, Germany), operating in parallel mode at an alternating frequency of 120 Hz (error at kΩ values < 0.5%). The tips of measuring probes were immersed in the donor and acceptor chamber, and filled with PBS (pH 7.4), as described previously [53,54]. Only skin samples with impedance > 3 kΩ were used for analysis, because these are values close to the electrical resistance of human skin [54]. 

The skin permeation test was performed for 24 h. At 1 h, 2 h, 3 h, 5 h, and 8 h after the start of the study, 1 mL of the acceptor fluid sample was collected and the acceptor chamber was refilled with fresh PBS solution. The last sample of the acceptor fluid was taken after 24 h. The concentration of phenolic acids and caffeine in the acceptor fluid was measured by the HPLC method. The cumulative mass (µg) of each phenolic acid and of caffeine was calculated based on the obtained concentration. The antioxidant activity of the acceptor fluid samples collected after completing the permeation study was also tested. After 24 h, the chambers were disassembled and the skin samples were removed and carefully rinsed in PBS (pH 7.4) [55]. The antioxidant activity and accumulation of selected phenolic acids, as well as caffeine, in the skin after permeation was assessed using a modified method described by Hag et al. [50]. A diffusion area (1 cm^2^) was cut out from each skin sample and dried at room temperature. These fragments were cut into small pieces, placed in 2 mL of methanol, and incubated for 24 h at 4 °C. Then, the skin samples were homogenized for 3 min using a homogenizer (IKA^®^T18 digital ULTRA TURRAX, Staufen, Germany), and the homogenate was centrifuged for 5 min at 3500 rpm. The supernatant was subjected to HPLC and spectrophotometric analyses, using pure methanol as a control. Accumulation of the phenolic acids and caffeine in the skin was calculated by dividing the amount of the substances remaining in the skin by the mass of the skin sample and was expressed as the mass of phenolic acid or caffeine per mass of the skin (μg·g^−1^). The antioxidant activity of the solution obtained after skin extraction was also assessed.

#### 4.2.4. pH Evaluation

The pH of both the fermented beverage and the unfermented control was determined by a pH meter (SCHOTT Instruments; SI Analytics Mainz, Mainz, Germany).

#### 4.2.5. Statistical Analysis

Results are presented as the mean ± standard deviation (SD). A one-way analysis of variance (ANOVA) was used. The significance of differences between individual kombuchas was evaluated with Tukey’s test (α < 0.05). Statistical calculations were done using Statistica 13.3 PL software (StatSoft, Kraków, Polska).

## 5. Conclusions

Several differences were observed between the results obtained for both kombuchas. GTK and BTK show similar antioxidant activity assessed by the DPPH method; however, in the case of GTK, higher activity assessed by FRAP and ABTS techniques and total polyphenol content were observed. However, the antioxidant activity and total polyphenol content in the acceptor fluid and skin extract after the permeation test were higher in the case of BTK. Moreover, HPLC analysis showed that BTK is characterized by a higher content of the tested phenolic acids and caffeine. Gallic acid and protocatechuic acid accumulated in the skin in similar amounts for both BTK and GTK. In the case of GTK, a higher accumulation of chlorogenic, caffeic, and coumaric acids was observed in the skin, while after the use of BTK a higher accumulation of m-hydroxybenzoic acid and caffeine was observed. HPLC analysis of the acceptor fluids after the permeation test showed higher content of protocatechuic, chlorogenic, and m-hydroxybenzoic acids in the case of GTK and gallic acid and caffeine in the case of BTK. The content of caffeic acid and coumaric acid in the acceptor fluids of both kombuchas was at a similar level. The cumulative permeation mass of caffeine during permeation testing was higher for BTK.

To sum up, the tested kombuchas prepared from black and green tea have high antioxidant potential, as well as containing phenolic acids and caffeine. They permeate the skin to a high degree and accumulate there, which means that their use in cosmetic products may have a positive effect on delaying the processes underlying skin aging. However, the use of kombucha in a specific cosmetic preparation should be preceded by an in-depth analysis in terms of the expected cosmetic effects, because other ingredients in the preparation, such as excipients, vehicles, or other active ingredients, may significantly affect, among other factors, its action, release, and ability to penetrate and accumulate in the skin.

## Figures and Tables

**Table 1:** The antioxidant activity and total polyphenol content (TPC) of green (GTK) and black (BTK) tea kombucha. Mean (±SD), n = 3, different letters indicate significant differences between the tested kombuchas; α = 0.05.

Type of Kombucha	DPPH		ABTS		FRAP	TPC
	(%RSA)	(mmol trolox·dm^−3^)	(%RSA)	(mmol trolox·dm^−3^)	(mmol FeSO_4_·dm^−3^)	(mg GA·dm^−3^)
GTK	87.08 ± 0.72 ^a^	0.82 ± 0.01 ^a^	93.88 ± 0.21 ^a^	3.43 ± 0.01 ^a^	8.87 ± 0.49 ^a^	195.84 ± 0.00 ^a^
BTK	87.68 ± 0.29 ^a^	0.83 ± 0.00 ^a^	71.01 ± 1.01 ^b^	2.59 ± 0.05 ^b^	3.36 ± 0.12 ^b^	188.13 ± 0.01 ^a^

**Table 2:** Phenolic acids and caffeine content in green and black tea kombucha. Mean (±SD), (n = 3); different letters indicate significant differences between the tested kombuchas; α = 0.05.

	Green Tea Kombucha (mg·dm^−3^)	Black Tea Kombucha (mg·dm^−3^)
gallic acid	32.84 ± 0.52 ^a^	49.22 ± 1.65 ^b^
protocatechuic acid	3.43 ± 0.19 ^a^	5.01 ± 0.40 ^b^
chlorogenic acid	5.13 ± 0.58 ^a^	7.60 ± 0.21 ^b^
caffeic acid	20.12 ± 0.46 ^a^	30.50 ± 0.39 ^b^
caffeine	102.87 ± 0.87 ^a^	165.49 ± 6.41 ^b^
m-hydroxybenzoic	10.96 ± 0.78 ^a^	19.15 ± 0.56 ^b^
coumaric acid	12.31 ± 0.31 ^a^	19.29 ± 0.69 ^b^

**Table 3:** Mean (±SD) antioxidant activity and TPC of the kombuchas applied on the skin, extract of skin extraction and acceptor fluid after 24-h permeation (n = 3); different letters indicate significant differences between the tested kombuchas; α = 0.05.

	Green Tea Kombucha		Black Tea Kombucha	
	DPPH (mmol trolox·dm^−3^)	TPC (mg GA·dm^−3^)	DPPH (mmol trolox·dm^−3^)	TPC (mg GA·dm^−3^)
kombucha applied to the skin	0.82 ± 0.01 ^a^	195.84 ± 0.00 ^a^	0.83 ± 0.00 ^a^	188.13 ± 0.01 ^a^
extract of skin extraction after 24-h permeation	0.31 ± 0.03 ^a^	88.27 ± 0.01 ^a^	0.40 ± 0.01 ^b^	104.02 ± 0.01 ^b^
extract of skin extraction after 24-h permeation (%)	37.80	45.07	48.19	55.29
acceptor fluid after 24-h permeation	0.05 ± 0.01 ^a^	24.60 ± 0.01 ^a^	0.07 ± 0.02 ^a^	28.28 ± 0.01 ^a^
acceptor fluid after 24-h permeation (%)	6.09	12.56	8.43	15.03

**Table 4:** Mean (±SD) content of phenolic acids and caffeine in acceptor fluid and extract obtained after 24-h permeation study in tested kombuchas, (n = 3); different letters indicate significant differences between the tested kombuchas; α = 0.05.

	Green Tea Kombucha				Black Tea Kombucha			
	Cumulating in the Skin		Acceptor Fluid after 24-h Permeation		Cumulating in the Skin		Acceptor Fluid after 24-h Permeation	
	(µg·g^−1^ of Skin)	(%)	(µg)	(%)	(µg·g^−1^ of Skin)	(%)	(µg)	(%)
gallic acid	123.26 ± 6.49 ^a^	38.47	4.58 ± 0.307 ^b^	1.74	126.87 ± 3.80 ^a^	26.44	6.50 ± 0.58 ^a^	1.65
protocatechuic acid	28.31 ± 1.87 ^a^	17.09	15.87 ± 0.59 ^a^	5.83	28.07 ± 1.21 ^a^	5.75	6.46 ± 1.11 ^b^	1.59
chlorogenic acid	12.31 ± 0.81 ^a^	24.68	3.89 ±0.58 ^a^	9.48	3.19 ± 0.49 ^b^	4.49	2.19 ± 0.10 ^b^	3.61
caffeic acid	42.60 ± 1.57 ^a^	21.75	2.02 ± 0.13 ^a^	1.00	19.23 ± 2.11 ^b^	6.73	2.29 ± 0.14 ^a^	0.75
caffeine	304.84 ± 18.46 ^a^	30.43	36.01 ± 3.73 ^b^	4.38	449.09 ± 36.14 ^b^	29.00	62.68 ± 1.18 ^a^	4.73
*m*-hydroxybenzoic acid	29.05 ± 3.42 ^b^	27.13	8.50 ± 0.94 ^a^	9.69	143.59 ± 17.82 ^a^	76.95	2.23 ± 0.11 ^b^	1.46
coumaric acid	30.88 ± 2.17 ^a^	25.75	5.93 ± 0.76 ^a^	6.02	20.26 ± 1.02 ^b^	11.25	5.74 ± 0.25 ^a^	3.71

**Table 5:** Characterization of the tested material based on the labels on the packaging.

Type of Kombucha	Composition	Energy Value per 100 mL	Fat, Including Saturated Acids (g)	Carbohydrate, Including Sugars (g)	Protein (g)	Salt (g)	Alcohol (%)
black tea (BTK)	Water, black tea, cane sugar, live bacteria cultures, hibiscus (10%)	26 kcal	<0.1 (<0.1)	6.3 (6.3)	<0.3	0.0062	<1.2
green tea (GTK)	Water, green tea, cane sugar, live bacteria cultures	26 kcal	<0.1 (<0.1)	6.3 (6.3)	<0.3	0.0062	<1.2

## Data Availability

Data are contained within the article.
